# Intranuclear inclusions of polyQ-expanded ATXN1 sequester RNA molecules

**DOI:** 10.3389/fnmol.2023.1280546

**Published:** 2023-12-06

**Authors:** Ioannis Gkekas, Aimilia-Christina Vagiona, Nikolaos Pechlivanis, Georgia Kastrinaki, Katerina Pliatsika, Sebastian Iben, Konstantinos Xanthopoulos, Fotis E. Psomopoulos, Miguel A. Andrade-Navarro, Spyros Petrakis

**Affiliations:** ^1^Centre for Research and Technology Hellas, Institute of Applied Biosciences, Thessaloniki, Greece; ^2^Laboratory of Pharmacology, School of Pharmacy, Faculty of Health Sciences, Aristotle University of Thessaloniki, Thessaloniki, Greece; ^3^Faculty of Biology, Johannes Gutenberg University Mainz, Mainz, Germany; ^4^Aerosol and Particle Technology Laboratory, Centre for Research and Technology Hellas, Chemical Process and Energy Resources Institute, Thessaloniki, Greece; ^5^Department of Dermatology and Allergic Diseases, University of Ulm, Ulm, Germany

**Keywords:** spinocerebellar ataxia type 1, ataxin-1, intranuclear inclusion bodies, RNA sequestration, RNA-seq, protein–protein interaction network, ribosome

## Abstract

Spinocerebellar ataxia type 1 (SCA1) is an autosomal dominant neurodegenerative disease caused by a trinucleotide (CAG) repeat expansion in the ATXN1 gene. It is characterized by the presence of polyglutamine (polyQ) intranuclear inclusion bodies (IIBs) within affected neurons. In order to investigate the impact of polyQ IIBs in SCA1 pathogenesis, we generated a novel protein aggregation model by inducible overexpression of the mutant ATXN1(Q82) isoform in human neuroblastoma SH-SY5Y cells. Moreover, we developed a simple and reproducible protocol for the efficient isolation of insoluble IIBs. Biophysical characterization showed that polyQ IIBs are enriched in RNA molecules which were further identified by next-generation sequencing. Finally, a protein interaction network analysis indicated that sequestration of essential RNA transcripts within ATXN1(Q82) IIBs may affect the ribosome resulting in error-prone protein synthesis and global proteome instability. These findings provide novel insights into the molecular pathogenesis of SCA1, highlighting the role of polyQ IIBs and their impact on critical cellular processes.

## Introduction

Spinocerebellar ataxia type 1 (SCA1) is a rare neurodegenerative disease belonging to the group of polyglutamine (polyQ) diseases. It is caused by trinucleotide (CAG) repeat expansions in the *ATXN1* gene resulting in the production of an abnormal polyQ tract in the ataxin-1 (ATXN1) protein (Chung et al., 1993). The length of the polyQ tract directly correlates with the age of onset and the severity of the disease (Orr et al., 1993). In healthy individuals, it typically ranges from 4 to 39 glutamines whereas in SCA1 patients it is significantly expanded, ranging from 40 to 83 glutamines. SCA1 is characterized by cerebellar atrophy, as well as degeneration of the brainstem and the spinal cord (Guerrini et al., 2004; Döhlinger et al., 2008; Pedroso and Barsottini, 2013). Disease pathology involves loss of Purkinje cells and reduction in the number of granule cells or other neuronal populations but may also extend beyond the cerebellum affecting other brain regions (Coffin et al., 2023).

ATXN1 is involved in transcriptional regulation and RNA splicing through its interaction with transcription factors and RNA-binding proteins (Mizutani et al., 2005; Tsuda et al., 2005; Zhang et al., 2020; Coffin et al., 2023). However, expansion of the polyQ tract in the mutant protein results in the formation of intranuclear inclusion bodies (IIBs), a striking feature of SCA1 (Cummings et al., 1999). ATXN1 IIBs sequester a variety of proteins, including proteasome and ribosome subunits, ubiquitin-related enzymes, kinases and heat-shock proteins, potentially impairing critical cellular functions and contributing to SCA1 pathology (Laidou et al., 2020). ATXN1 is also characterized by RNA-binding activity and may interact with multiple RNA targets. This feature is partially affected by the length of the polyQ tract (Yue et al., 2001; Chen et al., 2022); however, the extent to which this mechanism contributes to the pathogenesis of SCA1 remains to be elucidated.

Numerous findings support the notion that pathogenic protein inclusions contain RNA. RNA sequences have been found in inclusions derived from the hippocampus of Alzheimer’s disease (AD) patients and their number was significantly enriched compared to preparations from healthy individuals (Shmookler Reis et al., 2021). Furthermore, tau inclusions, detected in AD and Pick’s disease exhibit positive staining for RNA and are enriched in small nuclear and nucleolar RNAs (snRNAs and snoRNAs) (Ginsberg et al., 1997, 1998; Lester et al., 2021). Although significant research efforts have been made so far, the exact molecular composition and structure of protein inclusions associated with neurodegeneration remain poorly understood. The heterogeneity and complexity of these inclusions, along with the lack of efficient protocols for their isolation, present substantial challenges in characterizing their precise molecular organization.

Here, we generated human neuroblastoma SH-SY5Y cells with inducible overexpression of the SCA1-causing *ATXN1(Q82)* isoform and developed a protocol for the efficient isolation of polyQ-expanded ATXN 1 IIBs. Fourier-transform infrared spectroscopy (FTIR) indicated that these IIBs are enriched in RNA, a finding that was further validated by RNA-seq. Protein interaction network (PIN) analysis aiming to identify perturbed cellular processes in SCA1 cells, indicated that the sequestration of critical RNA transcripts in ATXN1(Q82) IIBs may affect the ribosome resulting in error-prone protein synthesis. The identification of RNA molecules within polyQ-expanded ATXN1 IIBs could provide valuable insights for the molecular mechanisms underlying SCA1 disease pathology.

## Materials and methods

### Generation of Tet-On YFP-ATXN1(Q82) and Venus SH-SY5Y cells

Human neuroblastoma SH-SY5Y cells were cultured in RPMI 1640 medium supplemented with 10% fetal bovine serum (FBS, Biowest, France) and penicillin- streptomycin (1X, Biowest, France) at 37°C in a humidified atmosphere containing 5% CO_2_. Tet-On YFP-ATXN1(Q82) SH-SY5Y cells were generated as previously described (Laidou et al., 2020). In brief, cells were seeded in a 6-well plate (2 × 10^5^ cells per well) and transfected with a mixture of pT2-CMV/TetO_2_-YFP-ATXN1(Q82), pT2-TetR-neo*^R^* (Laidou et al., 2020) and pCMV(CAT)T7-SB100 (Mátés et al., 2009) transposon plasmids at a 7:2:1 ratio using Xfect reagent (Clontech, USA). Venus SH-SY5Y cells were generated by transfection with pT2-GAGGS-Venus-neo*^R^* transposon (Dori et al., 2017) and pCMV(CAT)T7-SB100 plasmids at a 5:1 ratio. Transfected cells were selected at day 4 post-transfection using 200 ug/mL G418 (InvivoGen, USA). For transgene induction, doxycycline (Dox, 2 ug/mL, Sigma-Aldrich, USA) was added to the culture medium and replenished every 48 h. Genetically modified cells were differentiated into neuron-like cells by culturing for 7 days in medium containing 10 μM retinoic acid (RA).

### Fluorescence microscopy

Tet-On YFP-ATXN1(Q82) SH-SY5Y cells were seeded in a 24-well plate and cultured in the presence of Dox. Cells were fixed with 4% formaldehyde PBS for 10 min and permeabilized for 10 min with 0.1% Triton-X 100 (Sigma-Aldrich, USA) PBS. Nuclei were stained with DAPI for 5 min at room temperature. Fluorescent cells were observed in a ZOE Fluorescent cell imager equipped with three fluorescence channels and an integrated digital camera (Bio-Rad, USA).

### Flow cytometry

Cells expressing the *YFP-ATXN1(Q82)* transgene were measured on a CYTEK NL-CLC flow cytometer (CYTEC, USA). For each sample, 30,000 events were recorded with a rate of 5,000 events per second. Data was acquired and analyzed with the SpectroFlo software.

### Filter retardation assay

Cell extracts were mixed with an equal volume of a 4% SDS (AppliChem, Denmark), 100 mm DTT solution. Samples were heated at 95°C for 10 min, diluted with 100 uL 0.2% SDS and filtered through a 0.2 um cellulose acetate membrane (Whatman, Merck). SDS-resistant inclusions retained on the membranes were detected using the anti-ATXN1 SA4645 antibody (1:1,000) (Petrakis et al., 2012).

### Isolation of polyQ protein inclusions

PolyQ protein inclusions were purified from technical triplicates of Tet-On YFP-ATXN1(Q82) SH-SY5Y at day 5 post-induction. Cells (4 × 10^6^) were harvested by trypsin-EDTA (Biowest, France), collected by centrifugation at 1,000 *g* for 5 min and washed twice with PBS. The resulting cell pellet was resuspended in hypotonic buffer containing 0.5% Triton-X 100 PBS and incubated on ice for 20 min. Intact nuclei were collected after centrifugation at 12,000 *g* for 10 min at 4°C. The resulting supernatant containing the cytoplasmic fraction was collected and stored at −20°C. The nuclear pellet was resuspended in RIPA buffer supplemented with protease/phosphatase inhibitors (Thermo Fisher Scientific, Denmark) and benzonase (Calbiochem-Novagen, USA) and incubated on ice for 40 min. PolyQ inclusions were purified from the nuclear fraction using an Amicon Ultra-0.5 concentrator with a 100 kDa molecular weight cut-off (Merck) and concentrated to 20 uL.

### Immunoblotting

Samples from cells lysed in RIPA buffer containing protease/phosphatase inhibitors and benzonase, and cytoplasmic or nuclear fractions were resolved in a 10% SDS-PAGE and transferred to polyvinylidene difluoride (PVDF) membranes (Thermo Fisher Scientific, Denmark). Protein bands were detected with antibodies against ataxin-1 (SA4645), GFP (2956, Cell Signaling Technologies, USA), lamin A/C (4777, Cell Signaling Technologies, USA) and GAPDH (5171, Cell Signaling Technologies, USA) and visualized using either NBT/BCIP (AppliChem, Denmark) or Luminata Western HRP Chemiluminescence Substrate (Merck).

### FTIR spectroscopy

YFP-ATXN1(Q82) protein inclusions were seeded on MirrIR low-e-glass slides (Kevley Technologies, USA) and dried in ambient conditions. Then glass slides were measured by ATR-FTIR method at a Jasco spectrometer (Jasco FTIR-6700, Japan). Spectra were collected in the range of 4,000–400 cm^–1^ by 180 scans with 4 cm^–1^ resolution and analyzed using Spectra Manager 2.15.12 software (Jasco Corporation, Japan).

### RNA-sequencing

RNA was extracted from purified polyQ IIBs or control samples using TRIzol (Thermo Fisher Scientific, Denmark). The obtained RNA was quantified by NanoDrop (Thermo Fisher Scientific, Denmark) and its integrity was checked by Bioanalyzer 2100 (Agilent, USA). Sequencing libraries were prepared using the NEBNext Ultra™ II Directional RNA Library Prep Kit for Illumina (New England Biolabs, USA), according to manufacturer’s instructions. Library preparations were QC checked by electrophoresis (Bioanalyzer 2100, Agilent, USA) and quantified by Qubit Fluorometer (Thermo Fisher Scientific, Denmark). Paired-end sequencing was performed in a NextSeq 500 Illumina platform using the NextSeq 550 System Mid-Output Kit (Illumina). RNA-seq data were deposited in the European Nucleotide Archive (ENA) under accession number PRJEB64635.

### Data analysis and visualization

Quality control was performed based on the *FastQC* software with raw reads being trimmed and filtered using the *Trim Galore!* software suite. Trimmed reads were aligned against the *hg38* assembly of the human genome using the *HISAT2* protocol with an overall mapping rate ranging from 56.74 to 62.65% (Kim et al., 2019). Unmapped reads were later aligned against 45S, 18S, 5.8S, 5S rRNA, and tRNA resulting in a high mapping rate for 45S above 90%. The *Samtools* suite was used for the processing of the BAM files (Danecek et al., 2021). The final gene quantification (hg38) was performed using the *featureCounts* program (Liao et al., 2014). Subsequent data and statistical analysis were carried out in R v4.2.2.

Differential expression analysis was performed using the *DESeq2* package (Love et al., 2014). Genes with a *p*-value lower than 0.05 were considered significant. Enriched genes (logFC > 0) were used for downstream analyses and visualized in a volcano plot. Principal component analysis (PCA) was performed using the prcomp function on the significantly enriched genes. Samples were projected on the resulting space of the first two principal components (PC1, PC2). Functional analysis was performed using the *pathfinder* package against the gene ontology resource (Cellular Component and Biological Process terms) (Ulgen et al., 2019). Specific enriched terms were visualized using the *ggplot2* library.

### RT-qPCR

RT-qPCR was performed using Luna Universal One-Step RT-qPCR Kit (New England Biolabs, USA) in an AriaMx Real-Time PCR (qPCR) instrument (Agilent, USA). Primers were designed using NCBI Primer-BLAST or selected from the literature (Zhu et al., 2023) and their sequences are shown in Supplementary Table 1. The correct size of amplified RT-qPCR products was verified by electrophoresis in a 2% agarose gel. Data were analyzed by the 2^–ΔCt^ method using *GAPDH* as a housekeeping gene.

### Motif discovery

The transcript sequences (including 3′,5′ UTRs and introns) of genes were extracted from the Ensembl BioMart database, using the human genome assembly, GRCh38.p13 (Cunningham et al., 2022). Long sequences were split into 9,497 smaller overlapping fragments of 100nt to the adjacent regions. Transcripts were analyzed with the STREME tool of the MEME suite to discover patterns (ungapped motifs) that were enriched with respect to a control set generated by shuffling the input dataset (Bailey, 2021). The motif width was set between 3 and 30 nucleotides length. STREME applies a statistical test at *p*-value threshold < 0.05 to determine the enrichment of motifs in the input transcripts compared to the control set. The common motif sequences were visualized as logos using enoLOGOS. Identified motifs were scanned for putative binding sites in the CIS-BP RNA Database (Ray et al., 2013). Search was performed via the RNA Scan tool using the species parameter “Homo sapiens” and the motif model was set to the standard scoring system option which is position weight matrices (PWMs) – log-odds (Stormo, 1990). The log-odds threshold was set to its standard value of 6.

### Construction of the hPIN and embedding into the hyperbolic space

The human protein interaction network (hPIN) is a subset of release 2.3 of the Human Integrated Protein–Protein Interaction rEference (HIPPIE). The raw version of this network is available in the Download section of the HIPPIE database (Schaefer et al., 2012; Alanis-Lobato et al., 2017). After discarding self-interactions and extracting the network’s largest connected component (LCC), a hPIN was generated, consisting of 186,196 high-confidence interactions (score ≥ 0.71) between 15,587 proteins. Next, the hPIN was embedded into the two-dimensional hyperbolic plane using the R package “NetHypGeom” which implements the LaBNE + HM algorithm (Alanis-Lobato et al., 2016b). This algorithm combines manifold learning and maximum likelihood estimation to uncover the hidden geometry of complex networks (Papadopoulos et al., 2015; Alanis-Lobato et al., 2016a,b). The network was embedded into the H^2^ to infer the hyperbolic coordinates of each protein, with parameters γ = 2.97, *T* = 0.83 and *w* = 2π.

### SCA1 network in the hyperbolic space and clustering in the similarity dimension

The list of protein interactors was obtained from the HIPPIEv2.3 database and their position in the hPIN was determined (Schaefer et al., 2012; Alanis-Lobato et al., 2017). Groups of proteins were created in the network along the angular similarity dimension. To determine the start and the end of each group, proteins were sorted increasingly by their inferred angular coordinate (θ) and the difference between θ_*i*_ and θ_*i+*1_ was computed. The gap size (g) separating protein clusters produced sectors with a minimum of five components (*g* = 0.0346). Gene ontology (GO) (Ashburner et al., 2000) and KEGG pathway enrichment analysis (Kanehisa and Goto, 2000) for the proteins in each sector of the network was performed and enriched terms with *p*-value ≥ 0.05 were considered significant.

### Error-prone protein synthesis

Tet-On YFP-ATXN1(Q82) or Venus SH-SY5Y cells were cultured in white 96-well plates (4 × 10^4^ per well) and transfected with either wild-type or mutant (K529N) pCl-neo plasmids (500 ng per well) using Xfect reagent. The plasmids encode a dual reporter system, with the Renilla luciferase acting as the control reporter and the Firefly luciferase serving as the experimental reporter, both residing on a single pCI-neo plasmid. Luminescence produced by the Firefly and Renilla luciferases was quantified using a Dual-Glo assay kit (Promega, USA). Error-prone protein synthesis was determined based on the Firefly/Renilla ratio, as previously described (Alupei et al., 2018).

### BisANS assay

Cells were harvested by trypsinization and pelleted by centrifugation at 1,000 *g* for 5 min. The resulting pellet was resuspended in TNE buffer (50 mM Tris HCl, 100 mM NaCl, 1 mM EDTA) and cells were lysed by sonication (3 × 30 seconds at 25% amplitude). Cell extracts were centrifugated at 14,000 *g* for 20 min at 4°C. Protein concentration in the supernatant was quantified using NanoDrop (Thermo Fisher Scientific, Denmark). Next, 100 ug of protein was denatured by incubation in 2M urea for 2 h at room temperature. Then, BisANS dye (Cayman Chemical) was added (30 uM final concentration) and the mixture was incubated for 20 min to facilitate dye binding. Fluorescence was quantified with an excitation wavelength at 375 nm and emission data were recorded at 500 nm. Proteome instability was calculated by dividing the fluorescence signal after addition of BisANS to the background fluorescence of cells.

### Statistical analysis

Statistical analysis was performed using the GraphPad Prism software v9 (San Diego, USA). All experiments were performed in triplicates and results are shown as mean ± SD calculated by a *t*-test.

## Results

### Generation of Tet-On YFP-ATXN1(Q82) SH-SY5Y cells

**FIGURE 1:**
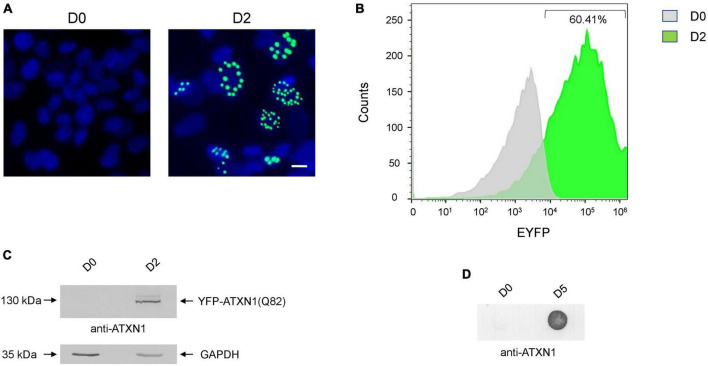
Generation of SH-SY5Y cells overexpressing the *YFP-ATXN1(Q82)* transgene under the Tet-On promoter. **(A)** Fluorescence microscopy of Tet-On YFP-ATXN1(Q82) SH-SY5Y cells in the absence (D0, left) or presence (D2, right) of Dox. Nuclei were stained with DAPI (blue) (scale bar = 10 μm) **(B)** Histogram plot depicting the percentage (%) of YFP-ATXN1(Q82) positive D0 (gray) or D2 (green) SH-SY5Y cells, as measured by flow cytometry. **(C)** Immunoblot for YFP-ATXN1(Q82) protein in extracts of uninduced (D0) and induced (D2) cells. GAPDH was used as a loading control. **(D)** Filter retardation assay for the detection of SDS-stable YFP-ATXN1(Q82) IIBs in extracts of D0 or D5 cells.

SH-SY5Y cells expressing *YFP-ATXN1(Q82)* under the control of the Tet-On promoter were generated using the sleeping beauty transposon technology. After G418 selection, genetically modified cells were characterized. Induction of transgene expression for 48 h resulted in the formation of IIBs of YFP-ATXN1(Q82) protein, whereas no fluorescence was observed in the absence of Dox (Figure 1A). Flow cytometry indicated that at least 60.41% of Tet-On YFP-ATXN1(Q82) SH-SY5Y cells were strongly YFP-positive 48 h post-induction (Figure 1B) while this percentage increased to 78% by D5 of induction (Supplementary Figures 1A, B). At this time point, two distinct populations were observed which may correspond to cells expressing low- or high-copy numbers of the transgene (Mátés et al., 2009). Production of YFP-ATXN1(Q82) was verified by SDS-PAGE and immunoblotting analysis in cell extracts of uninduced and induced cells. As expected, the recombinant protein was detected at 130 kDa only in extracts of induced cells whereas no such protein was detected in uninduced cells (Figure 1C). Immunoblotting using and anti-GFP antibody indicated that YFP-ATXN1(Q82) is not cleaved but is rather constantly produced as a fusion protein (Supplementary Figure 1C).

SCA1 is characterized by the formation of insoluble protein inclusions, mainly consisting of mutant ATXN1 (Zoghbi and Orr, 2009). The solubility of polyQ-expanded ATXN1 IIBs in Tet-On YFP-ATXN1(Q82) SH-SY5Y cells was assessed using a filter retardation assay. Insoluble inclusions of YFP-ATXN1(Q82) were readily immunodetected in extracts of Tet-On YFP-ATXN1(Q82) cells producing the mutant protein for 5 days; in contrast, no such inclusions were observed in uninduced cells (Figure 1D). Interestingly, fluorescent polyQ IIBs were also detected in neuron-like Tet-On YFP-ATXN1(Q82) SH-SY5Y cells differentiated in the presence of RA (Supplementary Figure 2A). These findings demonstrate that Tet-On YFP-ATXN1(Q82) SH-SY5Y cells accumulate insoluble IIBs of polyQ-expanded ATXN1, in consistency with other cellular models of SCA1 (Laidou et al., 2020).

### Isolation and characterization of insoluble IIBs of polyQ-expanded ATXN1

**FIGURE 2:**
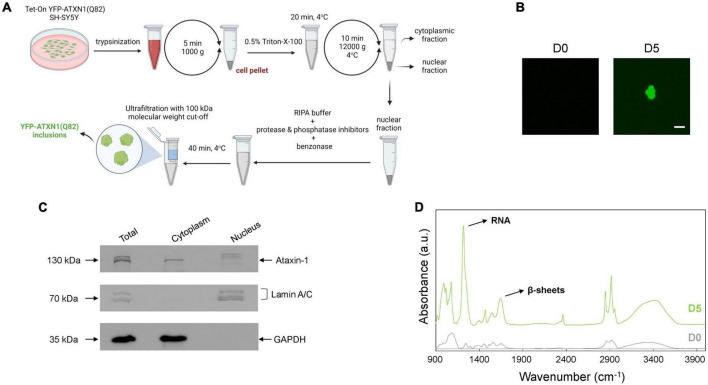
Isolation and characterization of YFP-ATXN1(Q82) IIBs. **(A)** Schematic representation of the protocol for the isolation of YFP-ATXN1(Q82) IIBs, including sample preparation, fractionation and purification steps. **(B)** Fluorescence microscopy of samples purified from uninduced, D0 (left) and induced, D5 cells (right), demonstrating the efficient isolation of YFP-ATXN1(Q82) IIBs (scale bar = 5 μM). **(C)** Immunoblots for YFP-ATXN1(Q82), lamin A/C (nuclear marker) and GAPDH (cytoplasmic marker) in whole-cell (total) extracts, cytoplasmic and nuclear fractions isolated from D5 cells. **(D)** FTIR absorption spectra of purified samples from D0 to D5 Tet-On YFP-ATXN1(Q82) SH-SY5Y cells (a.u.: arbitrary units). The D5 spectrum exhibits a prominent peak at 1,100 cm^–1^, indicating the presence of RNA and a peak at 1,640 cm^–1^, characteristic of proteins with a β-sheet conformation.

The precise cellular impact of ATXN1 inclusions remains poorly understood due to the difficulties associated with their isolation. Here, we developed a protocol for the isolation of insoluble IIBs from Tet-On YFP-ATXN1(Q82) SH-SY5Y cells. Insoluble YFP-ATXN1(Q82) IIBs were isolated from genetically modified cells at day 5 post-induction. The protocol consisted of discrete steps for: (1) the isolation of intact nuclei containing IIBs by centrifugation, (2) lysis of the pelleted nuclei and prolonged incubation with benzonase to remove residual nucleic acids, and (3) purification of insoluble IIBs using concentrators with high molecular-weight cut-off (Figure 2A). As shown in Figure 2B, this process reproducibly yielded large globular fluorescent inclusions of YFP-ATXN1(Q82), validating the effectiveness of the experimental protocol. Similarly, insoluble polyQ IIBs were isolated from neuron-like Tet-On YFP-ATXN1(Q82) SH-SY5Y cells at D5 post-induction (Supplementary Figure 2B). In contrast, application of the same protocol in preparations from control (D0) samples did not result in the isolation of similar structures.

Next, we evaluated the efficiency of the purification protocol. Equal amounts of cytoplasmic and nuclear extracts (Figure 2A) were analyzed by SDS-PAGE while whole-cell extracts served as control. In order to assess the purity of the fractions, marker proteins known to specifically localize in the nucleus (lamin A/C) or the cytoplasm (GAPDH) were detected by immunoblots. As expected, both marker proteins were detected in whole-cell extracts (total fraction), whereas lamin A/C was detected only in the nuclear fraction and GAPDH only in the cytoplasmic fraction. These results validate the efficient isolation of intact nuclei and the lack of contamination with cytoplasmic components. ATXN1 localizes in both the cytoplasm and the nucleus; therefore, recombinant YFP-ATXN1(Q82) was detected in both extracts. Interestingly, multiple bands were observed in whole-cell extracts from day 5 cells containing insoluble IIBs. The cytoplasmic fraction contained only a single band at approximately 130 kDa, corresponding to soluble YFP-ATXN1(Q82), whereas distinct bands corresponding to higher molecular weight, partially insoluble forms of YFP-ATXN1(Q82) protein were observed in the nuclear fraction (Figure 2C).

Given the well-known propensity of the pathological ATXN1 to adopt a beta-sheet conformation, we employed FTIR to obtain structural insights on YFP-ATXN1(Q82) IIBs. First, absorption spectra (400–4,000 cm^–1^) were collected from purified IIBs and control samples prepared from uninduced cells, using the previously established isolation protocol. This range of the FTIR spectrum includes the fundamental vibrations of the molecular bonds and provides information on the chemical composition and structure of the sample. Compared to control samples, the spectrum of YFP-ATXN1(Q82) IIBs indicated the presence of beta-sheets, as shown by a peak at 1,640 cm^–1^ corresponding to the amide I band. This observation is in agreement with a previous study showing that this peak characterizes the presence of beta-sheets in insoluble IIBs of YFP-ATXN1(Q82) (Laidou et al., 2020). Remarkably, a pronounced peak at approximately 1,100 cm^–1^, corresponding to ribonucleic acid (RNA), was also detected (Figure 2D), suggesting that these inclusions may contain RNA molecules. This peak was not detected in control samples prepared using the same IIB isolation protocol. Furthermore, the use of the endonucleolytic enzyme benzonase, which specifically degrades free DNA and RNA, excludes the possibility that this peak represents an artifact of the purification protocol or contamination with residual RNA. It rather reflects the presence of RNA, specifically associated with polyQ IIBs, that is protected from degradation by benzonase.

In order to validate this observation, we generated control overexpression SH-SY5Y cells stably producing Venus fluorescent protein, a variant of YFP (Nagai et al., 2002). Venus SH-SY5Y cells were uniformly fluorescent and produced the recombinant protein, as indicated by flow cytometry and immunoblotting (Supplementary Figure 3). These cells were further utilized for the isolation of RNA, according to the protocol described in Figure 2A. Extracted RNA was analyzed in a Bioanalyzer system providing a comprehensive view of RNA integrity and size distribution which indicated that no RNA can be isolated from Venus SH-SY5Y cells. In contrast, RNA with a similar pattern was isolated from polyQ IIBs of normal and neuron-like Tet-On YFP-ATXN1(Q82) SH-SY5Y cells (Supplementary Figure 2C).

### Identification of RNA molecules bound on IIBs of polyQ-expanded ATXN1

**FIGURE 3:**
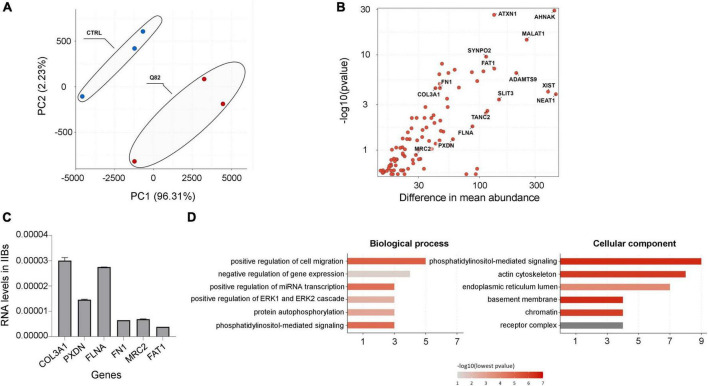
Sequencing of RNA transcripts enriched in polyQ IIBs. **(A)** PCA of sequenced RNA transcripts from polyQ IIBs (Q82, red) and control (CTRL, blue) samples. The plot shows the distribution of the samples based on the first two principal components. Percentages (%) indicate the proportion of variance per axis. **(B)** Volcano plot showing the 96 significantly enriched RNA transcripts in polyQ IIBs compared to CTRL preparations. **(C)** RT-qPCR validation for the enrichment of 6 randomly selected RNA transcripts in polyQ IIBs. **(D)** GO CC (upper panel) and BP (lower panel) enrichment analysis for the 96 enriched RNA transcripts. Bar color represents *p*-value ranging from red (most significant) to gray (least significant).

In order to determine the identity of the RNA molecules bound on insoluble polyQ IIBs, RNA was isolated from YFP-ATXN1(Q82) IIBs (Q82), followed by RNA sequencing. To ensure the reliability of the results and to rule out the possibility of detecting non-specific products or artifacts, RNA was also isolated and sequenced from control samples (CTRL) which do not contain any inclusions (Figure 2B). Principal component analysis (PCA) indicated distinct clustering of Q82 from CTRL samples, suggesting that the transcriptomes of these two groups were significantly different (Figure 3A). Computational analysis indicated 96 genes that were specifically enriched in polyQ IIBs compared to control samples (*p*-value < 0.05) (Supplementary Table 2). The top enriched genes, including *COL3A1*, *PXDN*, *FLNA*, *FN1*, and *MRC2* are shown in Table 1 and Figure 3B; their position in the volcano plot reflects their significance and magnitude of differential enrichment in polyQ IIBs. Furthermore, the presence of selected RNA transcripts (*COL3A1*, *PXDN*, *FLNA*, *FN1*, *MRC2*, and *FAT1*) in YFP-ATXN1(Q82) IIBs was validated by RT-qPCR (Figure 3C), whereas no such transcripts were detected in control preparations.

**TABLE 1:** Top enriched genes in YFP-ATXN1(Q82) IIBs.

Gene symbol	Difference in mean abundance	*p*-value
*NEAT1*	451.36	< 0.0001
*AHNAK*	438.16	< 0.0001
*XIST*	387.15	< 0.0001
*MALAT1*	253.84	< 0.0001
*ADAMTS9*	206.82	< 0.0001
*SLIT3*	147.02	< 0.0001
*FAT1*	134.08	< 0.0001
*ATXN1*	133.59	< 0.0001
*TANC2*	117.03	< 0.0001
*SYNP02*	114.16	< 0.0001
*FLNA*	87.16	< 0.001
*PXDN*	59.29	< 0.01
*FN1*	45.88	< 0.0001
*COL3A1*	42.02	< 0.0001
*MRC2*	28.58	< 0.05

Selection criteria were log_2_FC > 0 and *p*-value < 0.05.

Gene ontology (GO) analysis of the 96 identified genes revealed significant enrichment for various biological processes. These include positive regulation of cell migration and miRNA transcription and negative regulation of gene expression. Additionally, the analysis identified several enriched cellular components, including collagen-containing extracellular matrix, actin cytoskeleton, basement membrane and chromatin (Figure 3D).

We then sought to identify common motif sequences in enriched RNA transcripts using the STREME tool, which enables the identification of common ungapped motifs ranging from 3–30 nucleotide (nt) length in transcript sequences. To this end, we utilized the transcript sequences of the top-44 highly significant genes (adj. *p*-value < 0.05). Out of 65 computationally identified motifs, three of them ranging from 13 to 25 nucleotides were significantly identified (based on their E-value) in the majority of enriched transcripts. In detail, the first motif was present in 38 out of the 44 transcripts while the second and the third motifs were detected in 39 out of the 44 transcripts. Sequence logos of these motifs, along with their E-value and the list of genes in which they were identified are shown in Supplementary Figure 4 and Supplementary Table 3. Interestingly, these motifs were also identified as potential binding sites for proteins belonging to the PUF, RRM, and KH families (Supplementary Table 4).

### RNA sequestration on polyQ IIBs dysregulates protein complexes

**FIGURE 4:**
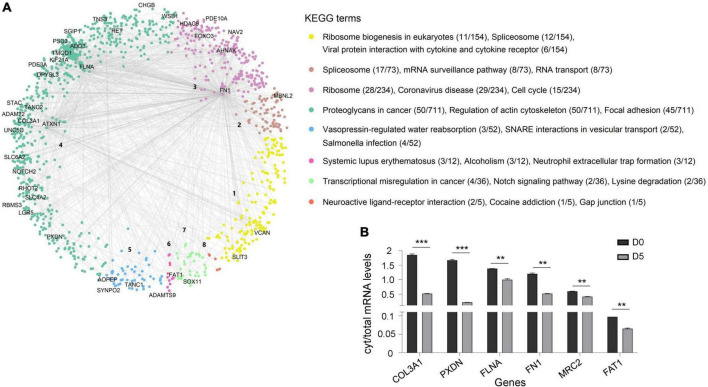
Dysregulated protein pathways due to the sequestration of RNA transcripts in polyQ IIBs. **(A)** SCA1 PIN illustrating the interactions of proteins corresponding to the RNA transcripts bound on polyQ IIBs (40 genes, adj. *p*-value < 0.05). Network proteins agglomerated into eight clusters; each were assigned a numeric identifier (1–8). KEGG pathway enrichment analysis indicates the overrepresented function of each cluster. The figure also shows the number of proteins associated with each KEGG term per total proteins of the cluster. **(B)** Comparative analysis of cytoplasmic/total mRNA ratio for enriched RNA transcripts in (D5) versus (D0) Tet-On YFP-ATXN1(Q82) SH-SY5Y cells (**p-value < 0.01, ***p-value < 0.001).

RNA transcripts are typically synthesized in the nucleus and then exported to the cytoplasm for translation into proteins. Therefore, we hypothesized that sequestration of RNA on polyQ IIBs might disturb synthesis and stoichiometry of proteins, resulting in dysregulation of relevant protein complexes. To determine dysregulated processes in Tet-On YFP-ATXN1(Q82) SH-SY5Y cells, we generated a hPIN using as input interactions of human proteins corresponding to the RNA transcripts bound on polyQ IIBs. The resulting network was embedded into the two-dimensional hyperbolic plane H^2^. Then, a list of proteins interacting with the products of the 40/44 most significant (adj. *p*-value < 0.05) protein coding genes was obtained; four RNA genes that did not encode a protein product were excluded from downstream analysis. The resulting SCA1 PIN consisted of 1,277 proteins, which were connected by 1,482 interactions (Figure 4A). The similarity component of the PS model (angular coordinates of nodes in H^2^) abstracts the characteristics that make a node similar to neighboring proteins participating in related biological process (Härtner et al., 2018; Vagiona et al., 2022). Functional modularity of the generated SCA1 PIN, was detected by the presence of big gaps between consecutive inferred angles. Therefore, protein components of the network agglomerated into 8 clusters. The overrepresented function of each cluster was determined by KEGG pathway enrichment analysis, highlighting the functional heterogeneity of the sectors and the similarity-based protein agglomeration. Indicatively, proteins of cluster 1 were found to participate in ribosome biogenesis while spliceosome, mRNA surveillance and RNA transport were the majorly enriched pathways of cluster 2. Proteins participating in cluster 3 and 4 were highly associated with the ribosome and focal adhesion (Figure 4A), in agreement with previous studies reporting perturbation of the protein synthesis machinery and extracellular matrix remodeling in SCA1 (Lee et al., 2011b; Vagiona et al., 2020).

We then sought to validate the partial cytoplasmic depletion of RNA transcripts corresponding to critical nodes of the dysregulated SCA1 PIN. Six genes were selected that either represented top-linked nodes (*FN1*, *FLNA*, and *FAT1* in clusters 3, 4 and 7, respectively) or were significantly enriched in ATXN1(Q82) IIBs (*COL3A1*, *PXDN*, and *MRC2*) (Supplementary Table 5). A comparative analysis of cytoplasmic/total mRNA levels was performed in Tet-On YFP-ATXN1(Q82) SH-SY5Y cells containing IIBs (D5) compared to control (D0) cells. As shown in Figure 4B, the cytoplasmic/total mRNA ratio of all selected genes was significantly decreased in D5 versus D0 cells, which did not contain inclusions. No such reduction was observed for the housekeeping gene *GAPDH* (Supplementary Figure 5). Additionally, total expression of *FN1*, *FLNA*, *FAT1*, *COL3A1*, *PXDN*, and *MRC2* genes was not down-regulated in D5 vs. D0 cells (Supplementary Figure 6), indicating the specificity of the previous findings. These results support our hypothesis that sequestration of RNA transcripts within polyQ-expanded ATXN1 IIBs affects their cytoplasmic availability and transcription, contributing to the functional dysregulation of specific protein complexes.

### PolyQ IIBs affect ribosomal activity

Multiple lines of evidence indicate that aggregation of mutant polyQ proteins disrupts the protein synthesis machinery (Laidou et al., 2020; Eshraghi et al., 2021). This notion is consistent with network analysis highlighting the dysregulation of the ribosome and its biogenesis in our cell model (clusters 1 and 3 of the hPIN shown in Figure 4A). To address this prediction, we assessed the elongation activity of RNA polymerase I, related to the pre-rRNA abundance of 5.8S/ITS2 and 28S/ETS, and the processing of the primary pre-rRNA transcript by measuring ITS1 levels. RT-qPCR analysis indicated significantly lower levels of all markers in Tet-On YFP-ATXN1(Q82) SH-SY5Y at D5 vs. Venus cells (Supplementary Figure 7); these disturbances might be indicative of problems with ribosome biogenesis and assembly, as described in the literature (Zhu et al., 2023).

**FIGURE 5:**
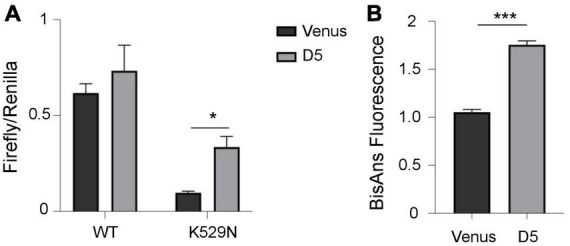
Error-prone protein synthesis and global proteome stability in cells producing mutant ATXN1. **(A)** Translational infidelity of induced (D5) Tet-On YFP-ATXN1(Q82) SH-SY5Y cells compared to Venus SH-SY5Y cells using a luciferase-based assay. **(B)** Proteome instability in induced (D5) Tet-On YFP-ATXN1(Q82) and Venus SH-SY5Y cells. Graphs indicate normalized fluorescence intensity after labeling with BisANS fluorescent dye. Error bars denote mean ± SD (*p-value < 0.05, ***p-value < 0.001).

Ribosomes decode the genetic information of mRNA and synthesize proteins by accurately matching each RNA codon to the corresponding amino acid. Errors due to ribosomal dysfunction may cause incorporation of incorrect amino acids into proteins, resulting in error-prone protein synthesis (Kapur and Ackerman, 2018). Therefore, we assessed translation accuracy in cells containing polyQ-expanded ATXN1 IIBs using a luciferase activity assay (Alupei et al., 2018). This assay detects the error rate of translation at the ribosome through a reporter system with a defined mutation in the active center of firefly luciferase (K529N). Correct translation due to the incorporation of asparagine results in minimum luciferase activity. However, incorrect, error prone translation re-activates luciferase activity by the random incorporation of the activating amino acid lysine. Activity of exogenous wild-type luciferase was similar in both induced (D5) and uninduced (D0) cells, as expected. However, the activity of mutant luciferase harboring the K529N point-mutation was partially restored only in D5 Tet-On YFP-ATXN1(Q82) SH-SY5Y (Supplementary Figure 8). Next, we compared mutant luciferase activity in D5 and Venus cells. Again, the activity of mutant luciferase was significantly higher in D5 cells, suggesting that the restoration of its function is not generically affected by overexpression of a random transgene in SH-SY5Y cells (Figure 5A).

Finally, we quantified global proteome stability in Tet-On YFP-ATXN1(Q82) SH-SY5Y by measuring the amount of exposed hydrophobic side chains, as previously described (Alupei et al., 2018). A significant elevation of misfolded proteins was observed in D5 vs. Venus cells (Figure 5B), suggesting that the proteome of these cells might be destabilized. Collectively, these results indicate the occurrence of error-prone protein synthesis in cells producing mutant ATXN1, directly affecting the quality of protein synthesis. In conclusion, here we show that sequestration of RNA transcripts within insoluble IIBs of polyQ-expanded ATXN1 affects various cellular processes and potentially, the assembly and function of the ribosome, the protein synthesis machinery.

## Discussion

### A cell model allowing the isolation of ATXN1(Q82) IIBs

Cell models provide valuable insights on the pathogenesis of neurodegenerative disorders, including SCA1. Induced pluripotent stem cells (iPSCs) derived from patient material enable the generation of disease-relevant cell types, facilitating detailed investigations and experimentation (Buijsen et al., 2018; Jin and Nan, 2023). However, reprogramming of somatic cells usually results in developmental reset and partial rejuvenation which may disrupt the aggregation of polyQ-expanded proteins in disease-specific neurons.

In an alternative approach for disease modeling, we overexpressed polyQ-expanded *ATXN1* in SH-SY5Y cells, which belong to the neuronal lineage, and demonstrate a relevant gene expression pattern. Tet-On YFP-ATXN1(Q82) SH-SY5Y cells allow the controlled expression of the transgene and most importantly, accumulate insoluble IIBs, which characterize SCA1. The ability to monitor the formation of IIBs in real-time further enhances the utility of this model for investigating SCA1 pathogenesis. Of note, our cell-model has certain limitations. Compared to patient-derived cellular models, it lacks the potential to differentiate into disease-relevant neuronal subtypes while overexpression of the polyQ-expanded *ATXN1* isoform may augment pathogenic phenotypes. On the other hand, our cell model can be used for the reproducible isolation of polyQ IIBs, also from neuron-like cells, enabling the study of their structure and composition. Existing protocols for the purification of pathogenic inclusions from human disease material are inefficient and lack reproducibility.

### Sequestration of RNA transcripts in polyQ IIBs affects important cellular processes

Compelling evidence substantiate the sequestration of RNA molecules within pathological aggregates associated with neurodegeneration. Indeed, protein aggregates characterizing Alzheimer’s and Pick’s disease were previously shown to contain RNA molecules (Ginsberg et al., 1997, 1998; Shmookler Reis et al., 2021). Furthermore, RNA binding drives the conformational change of tau and the oligomerization of TDP-43, associated with Alzheimer’s disease and FTD/ALS, respectively (McMillan et al., 2023; Pérez-Berlanga et al., 2023). Despite the well-established role of ATXN1 as an RNA-binding protein, the interplay between RNA and polyQ IIBs remains largely unknown. Here, we documented the sequestration of RNA transcripts within ATXN1 polyQ IIBs, highlighting the potential involvement of RNA in the pathogenesis of SCA1.

The presence of RNA molecules within polyQ IIBs raises intriguing questions regarding their composition and function. Motif analysis indicated shared sequences among enriched RNA transcripts suggesting their specific sequestration into polyQ IIBs. These motifs may be recognized by ATXN1 and be partially responsible for the global dysregulation of gene expression in SCA1 models. Interestingly, the top identified motif is strikingly similar to the binding site (UGUAAUC) of proteins belonging to the PUF family. It has been previously shown that *PUM1* is neuroprotective in cell models of SCA1 (Petrakis et al., 2012) while its haploinsufficiency causes SCA1-like neurodegeneration (Petrakis et al., 2012; Gennarino et al., 2015). PUM1 is also an RNA-binding protein and a known interactor of ATXN1 (Petrakis et al., 2012). Whether mutant ATXN1 and PUM1 proteins compete for binding to the same RNA molecules, along with the potential role of such a competition for SCA1 pathogenesis remains to be investigated.

Among the top statistically significant transcripts, we identified *NEAT1*, *AHNAK*, and *SYNPO2* as potential contributors to SCA1 neurodegeneration. *NEAT1* regulates the nuclear architecture and the formation of paraspeckles, nuclear bodies that are involved in the regulation of gene expression. Although the precise role of *NEAT1* in neurodegeneration remains elusive, its abnormal expression has been associated with various conditions, including amyotrophic lateral sclerosis (ALS), Alzheimer’s, Parkinson’s, and Huntington’s disease (Li and Wang, 2023). *AHNAK* encodes a nucleoprotein participating in intracellular calcium homeostasis, a process that is disrupted in polyQ diseases (Giacomello et al., 2013). *SYNPO2* is involved in the maintenance of the neuronal cytoskeleton, contributing to the stability of synaptic connections. Dysregulation of cytoskeletal dynamics and impaired synaptic function are common features in polyQ diseases (Lee et al., 2011a; Lim et al., 2017).

Gene ontology (GO) analysis of the 96 transcripts identified in polyQ IIBs provides novel insights for dysregulated cellular processes. Interestingly, computational analysis indicated that these genes participate in positive regulation of miRNA transcription, a regulatory process governing the production of microRNAs, which modulate gene expression; their dysregulation is a common feature of neurodegenerative diseases, including HD and SCA17 (Roshan et al., 2017; Catanesi et al., 2020; Tung et al., 2021). In terms of cellular components, this analysis highlighted the enrichment of genes associated with chromatin suggesting their potential involvement in chromatin remodeling and gene regulation, which may contribute to the dysregulation of gene expression in polyQ diseases.

We further hypothesized that RNA sequestration in polyQ IIBs may affect the function of critical protein complexes for SCA1 progression. Consequently, we focused on the ribosome which is frequently dysregulated in neurodegenerative diseases. We showed that sequestration of RNA transcripts in insoluble polyQ IIBs induces error-prone protein synthesis and enhances proteome instability, potentially affecting global protein synthesis, as previously documented for various polyQ diseases (Hübener et al., 2011). Our observation is in agreement with other studies reporting that mutant huntingtin stalls ribosomes and represses protein synthesis (Eshraghi et al., 2021) or indicating down-regulation of ribosomal proteins in SCA1 (Laidou et al., 2020).

ATXN1 is a widely expressed protein which normally, translocates between the cytoplasm and the nucleus where it binds RNA molecules. However, polyQ-expanded ATXN1 is not capable of nuclear export and its nuclear retention, along with its bound RNA molecules, may be partially responsible for its cytotoxicity (Irwin et al., 2005). Such pathogenic interplays may be widely common among neurodegenerative diseases. For example, the aggregation of TDP-43, an RNA-binding protein which is involved in ALS is modulated by its bound RNA molecules (Louka et al., 2020), highlighting its significance for disease progression. Concerning SCA1, neuronal vulnerability is due to complex mechanisms in different brain regions which are characterized by differential gene expression patterns (Coffin et al., 2023). The sequestration of RNA transcripts on polyQ IIBs, as shown here, may contribute to region-specific defects. In brief, different RNA transcripts may be sequestered on polyQ IIBs and trapped to the nucleus in the various brain regions. The impact of such events in SCA1 pathogenesis remains to be investigated.

## Data availability statement

The datasets presented in this study can be found in online repositories. The names of the repository/repositories and accession number(s) can be found in this article/Supplementary material.

## Author contributions

IG: Conceptualization, Methodology, Validation, Visualization, Writing – original draft. A-CV: Conceptualization, Formal analysis, Visualization, Writing – original draft. NP: Conceptualization, Formal analysis, Visualization, Writing – review and editing. GK: Conceptualization, Methodology, Visualization, Writing – original draft. KP: Methodology, Formal analysis, Visualization. SI: Methodology, Writing – review and editing. KX: Conceptualization, Writing – review and editing. FP: Conceptualization, Formal analysis, Writing – review and editing. MA-N: Conceptualization, Formal analysis, Writing – review and editing. SP: Conceptualization, Methodology, Formal analysis, Investigation, Supervision, Writing – original draft.
