# Supplementary material for: Intranuclear inclusions of polyQ-expanded ATXN1 sequester RNA molecules
Source: Front Mol Neurosci. 2023 Dec 6;16:1280546. doi: 10.3389/fnmol.2023.1280546 (PMC10730666; doi:10.3389/fnmol.2023.1280546)
Supplement: Supplementary file 1 [file Data_Sheet_1.PDF]

Supplementary Material

Supplementary figure legends

Supplementary Figure 1

(A) Fluorescence microscopy of induced (D5) Tet-On YFP-ATXN1(Q82) SH-SY5Y cells. Nuclei were stained with DAPI (blue) (scale bar = 15 µm). **(B)** Flow cytometry (histogram plot) of uninduced (grey) and induced (D5) Tet-On YFP-ATXN1(Q82) cells. **(C)** Immunoblot for YFP-ATXN1(Q82) protein in extracts of uninduced (D0) and induced (D5) cells using an anti-GFP antibody.

Supplementary Figure 2

(A) Merged fluorescence and brightfield microscopy of differentiated Tet-On YFP-ATXN1(Q82) SH-SY5Y cells in the presence (D5) of Dox (scale bar = 40 µm). **(B)** Fluorescence microscopy of purified IIBS from differentiated induced (D5) cells (scale bar = 5 µm). **(C)** Electrophoretic pattern of RNA samples isolated from un-differentiated (Lane 1), differentiated (Lane 2) D5 Tet-On YFP-ATXN1(Q82) or Venus (Lane 3) SH-SY5Y cells using the protocol described in Figure 2A.

Supplementary Figure 3

Generation of SH-SY5Y cells stably producing Venus protein. **(A)** Fluorescence microscopy of Venus SH-SY5Y cells. Nuclei were stained with DAPI (blue) (scale bar = 50 µm). **(B)** Flow cytometry (histogram plot) of untransfected (control, grey) and Venus (green) SH-SY5Y cells. **(C)** Immunoblot for Venus in protein extracts of control and Venus SH-SY5Y cells. GAPDH was used as a loading control.

Supplementary Figure 4

Top three sequence motifs identified in RNA transcripts enriched within YFP-ATXN1(Q82) IIBs.

Supplementary Figure 5

Comparative analysis of cytoplasmic/total mRNA ratio for the housekeeping gene GAPDH in (D5) versus (D0) Tet-On YFP-ATXN1(Q82) SH-SY5Y cells.

Supplementary Figure 6

Bar graphs indicating the expression levels of **(A)** COL3A1, FN1, MRC2 and **(B)** PXDN, FLNA, FAT1 genes in D0 and D5 Tet-On YFP-ATXN1(Q82) SH-SY5Y cells.

Supplementary Figure 7

RT-qPCR analysis for **(A)** ITS-1 levels, **(B)** 5.8S+ITS2 and **(C)** 28S/ETS pre-rRNA abundance in induced (D5) Tet-On YFP-ATXN1(Q82) and Venus SH-SY5Y cells. GAPDH was used as housekeeping gene. Error bars denote mean ±SD (* p-value < 0.05).

Supplementary Figure 8

Translational infidelity of induced (D5) Tet-On YFP-ATXN1(Q82) SH-SY5Y cells compared to uninduced (D0) cells using a luciferase-based assay. Error bars denote mean ± SD (* p-value < 0.05).

Supplementary Figure 9

Original Western blots of **(A)** Figure 1C and **(B)** Figure 2C.

Supplementary Tables Legends

Supplementary Table 1. Primer sequences used in RT-qPCR experiments.

Supplementary Table 2. List of genes corresponding to enriched RNA transcripts in polyQ IIBs. The table includes gene name, log2FC and p-value. It also shows the normalized expression levels in preparations from control (CTRL) and Q82 IIBs along with the difference in the mean abundance between the two sample sets.

Supplementary Table 3. Motif sequences identified in RNA transcripts bound on polyQ inclusions. The table shows the sequence and length of the motif, the statistical significance (E-value), the name of the corresponding gene, the length of the RNA transcript and the number of RNA fragments which contain the motif.

Supplementary Table 4. Comparison of the identified motifs with known motifs recognized by other RNA-binding (RBD) proteins. The table contains information on other RBD proteins (protein name, protein ID, gene ID, family classification) which partially recognize the motifs shown in Supplementary Table 3. It also shows the common sequence and its position in the identified motifs of Supplementary Table 3. Results with a score of at least 6 (standard threshold) were considered significant.

Supplementary Table 5. List of proteins in the SCA1 hPIN. List of proteins in the SCA1 PIN, identified by a unique UniprotKB ID and gene name. The position of each protein in the hyperbolic disc is indicated by the r and θ values. For each protein, the table shows the degree of connectivity (defined as the number of direct interactions with other proteins in the network) and the assignment into a network cluster.
