# Supplementary material for: Intranuclear inclusions of polyQ-expanded ATXN1 sequester RNA molecules
Source: Front Mol Neurosci. 2023 Dec 6;16:1280546. doi: 10.3389/fnmol.2023.1280546 (PMC10730666; doi:10.3389/fnmol.2023.1280546)
Supplement: Supplementary file 2 [file Presentation_1.PPTX]

## Slide 1
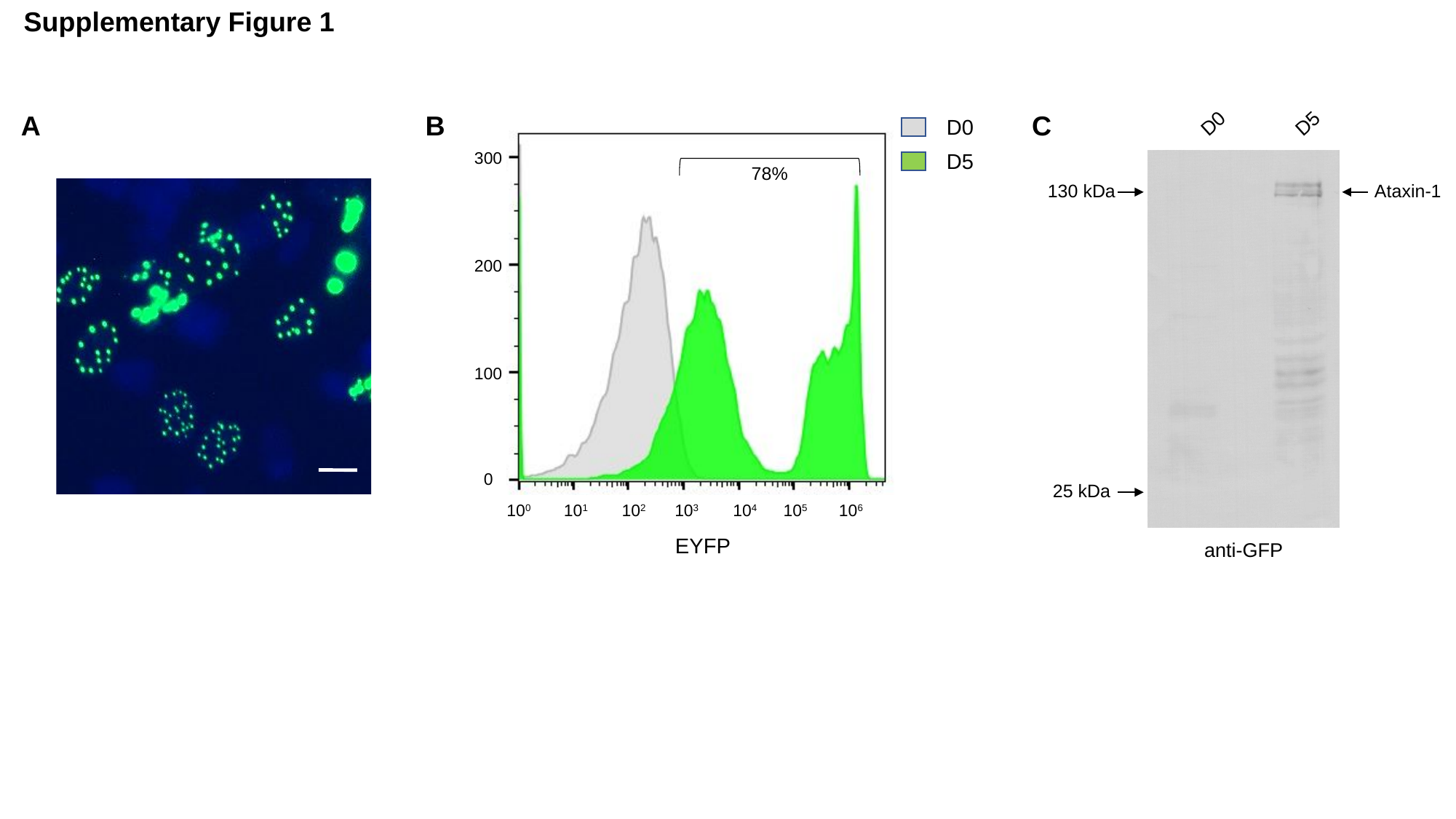

Supplementary Figure 1
A
B
C
D0
D5
130 kDa
Ataxin-1
25 kDa
anti-GFP
D0
300
D5
78%
200
100
0
100
101
102
103
104
105
106
EYFP

## Slide 2
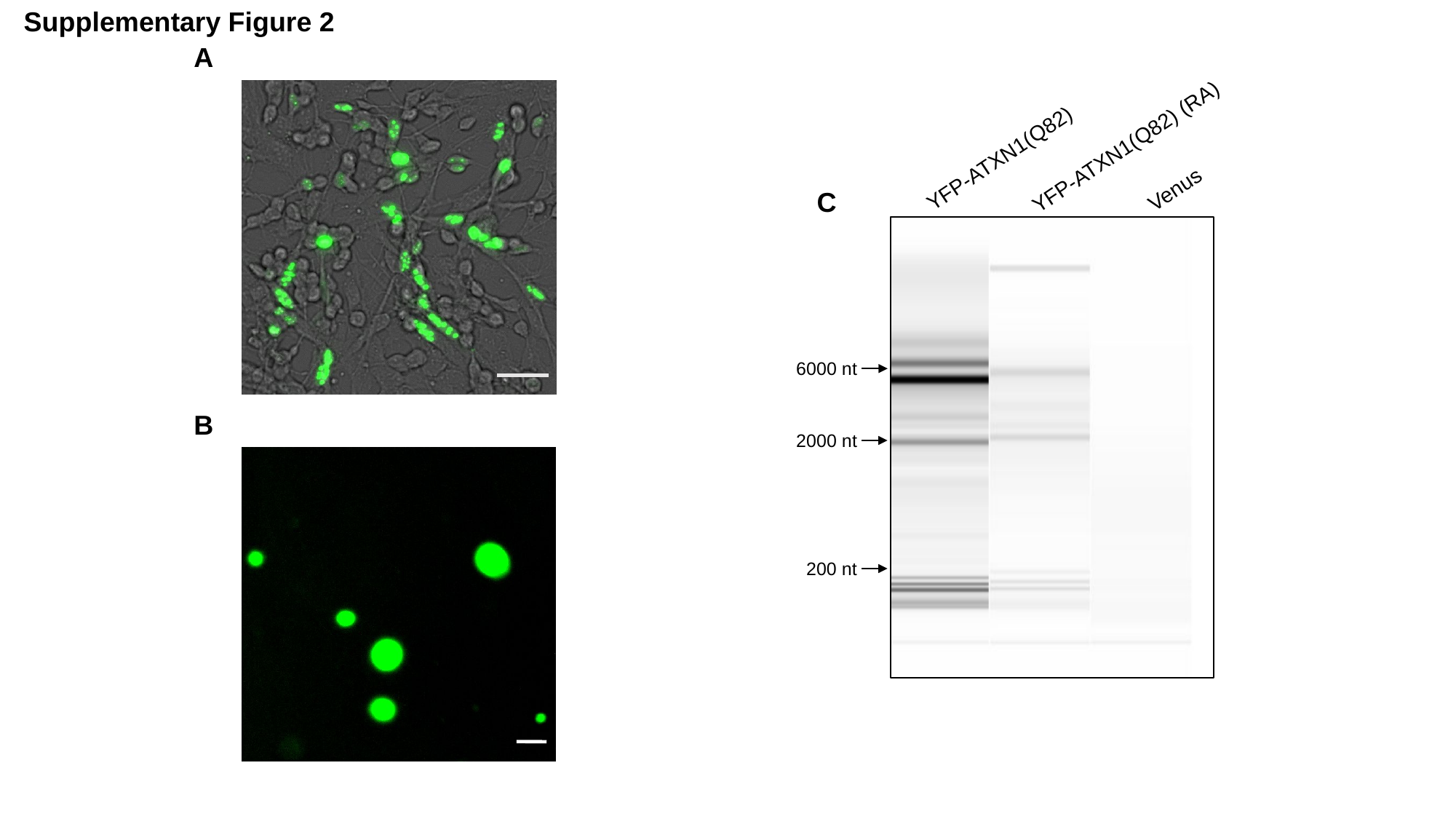

Supplementary Figure 2
A
YFP-ATXN1(Q82) (RA)
YFP-ATXN1(Q82)
Venus
C
6000 nt
B
2000 nt
200 nt

## Slide 3
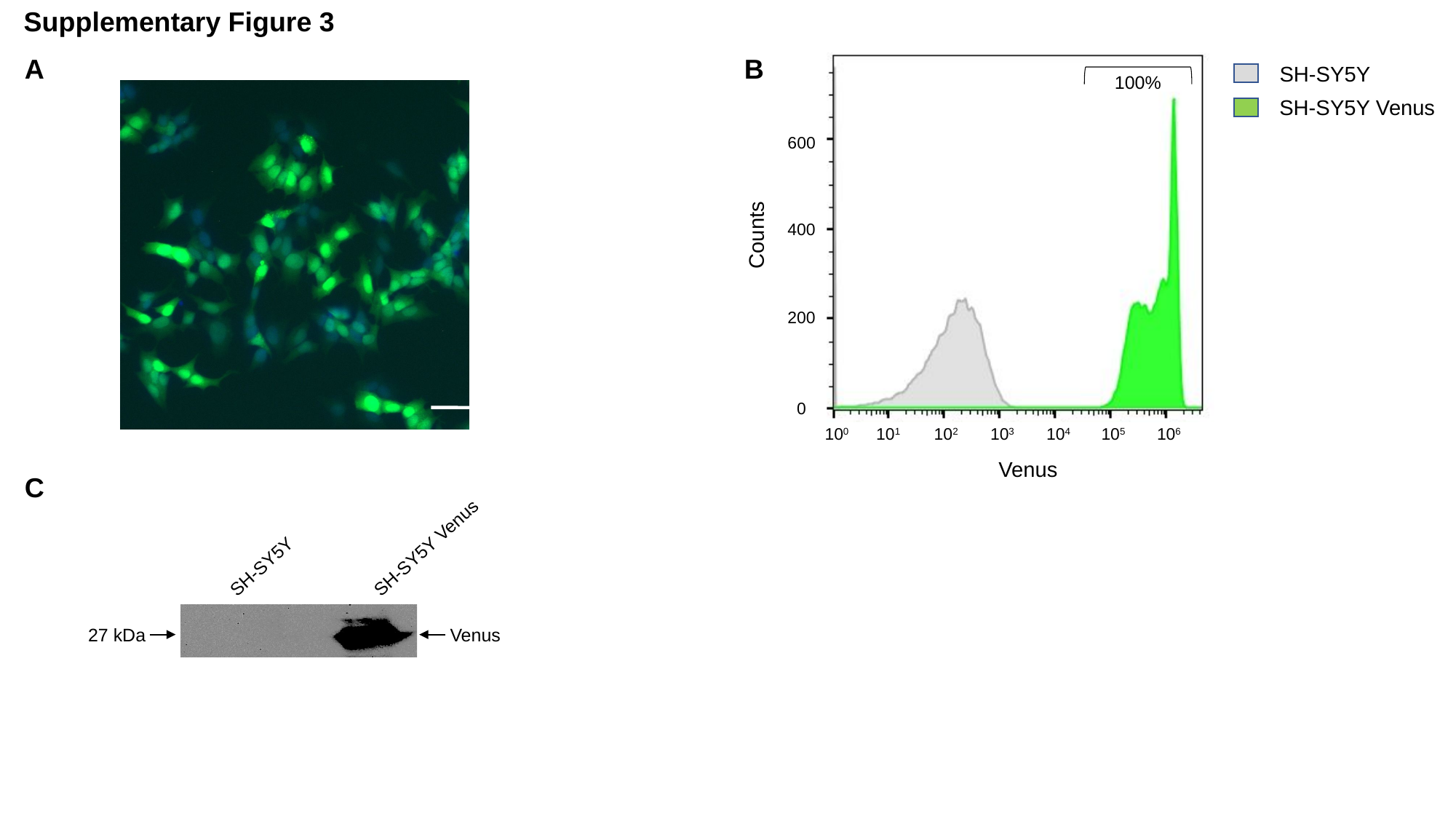

Supplementary Figure 3
A
B
SH-SY5Y
SH-SY5Y Venus
600
400
Counts
200
0
100
101
102
103
104
105
106
Venus
100%
C
SH-SY5Y Venus
SH-SY5Y
27 kDa
Venus

## Slide 4
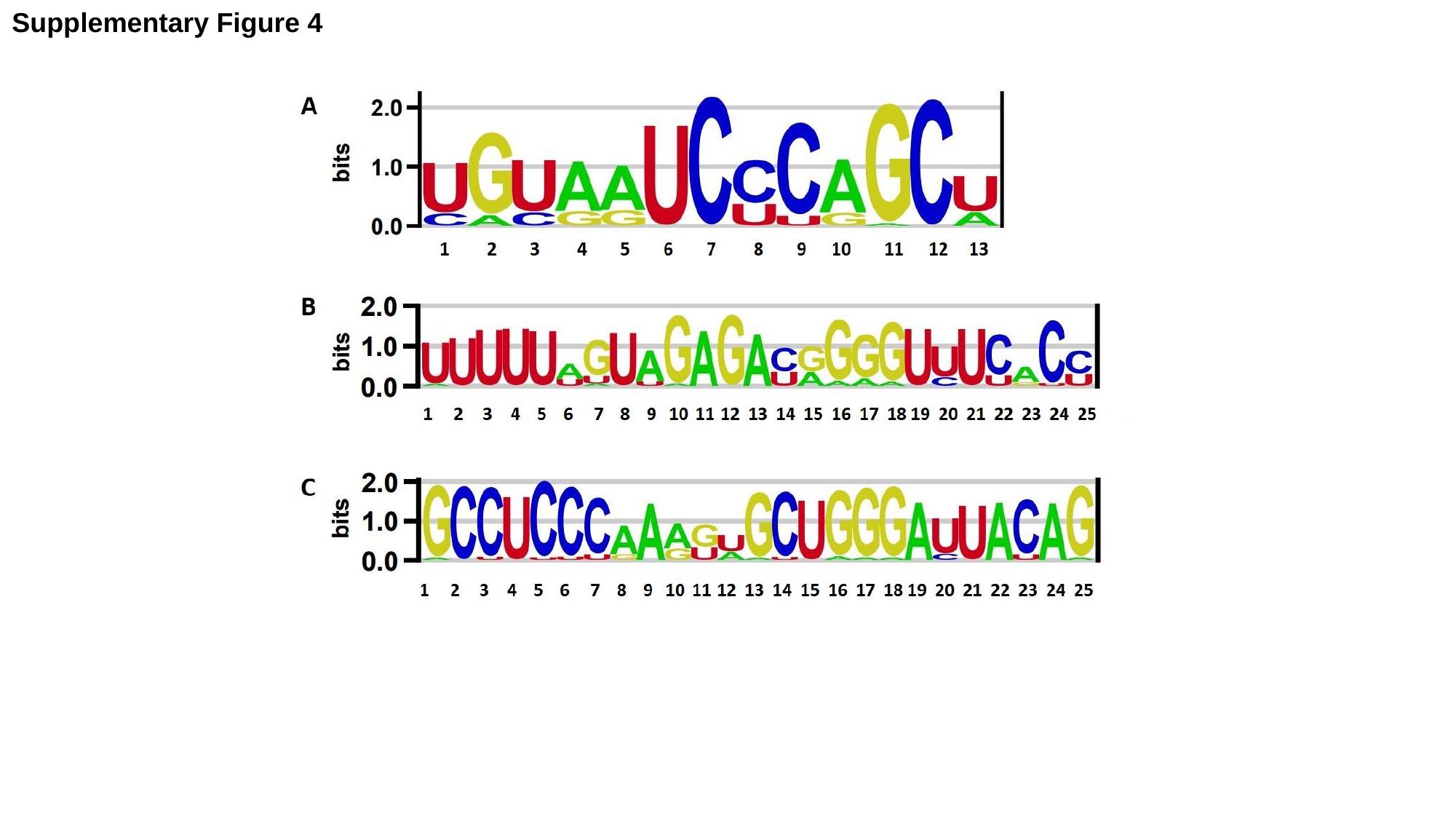

Supplementary Figure 4

## Slide 5
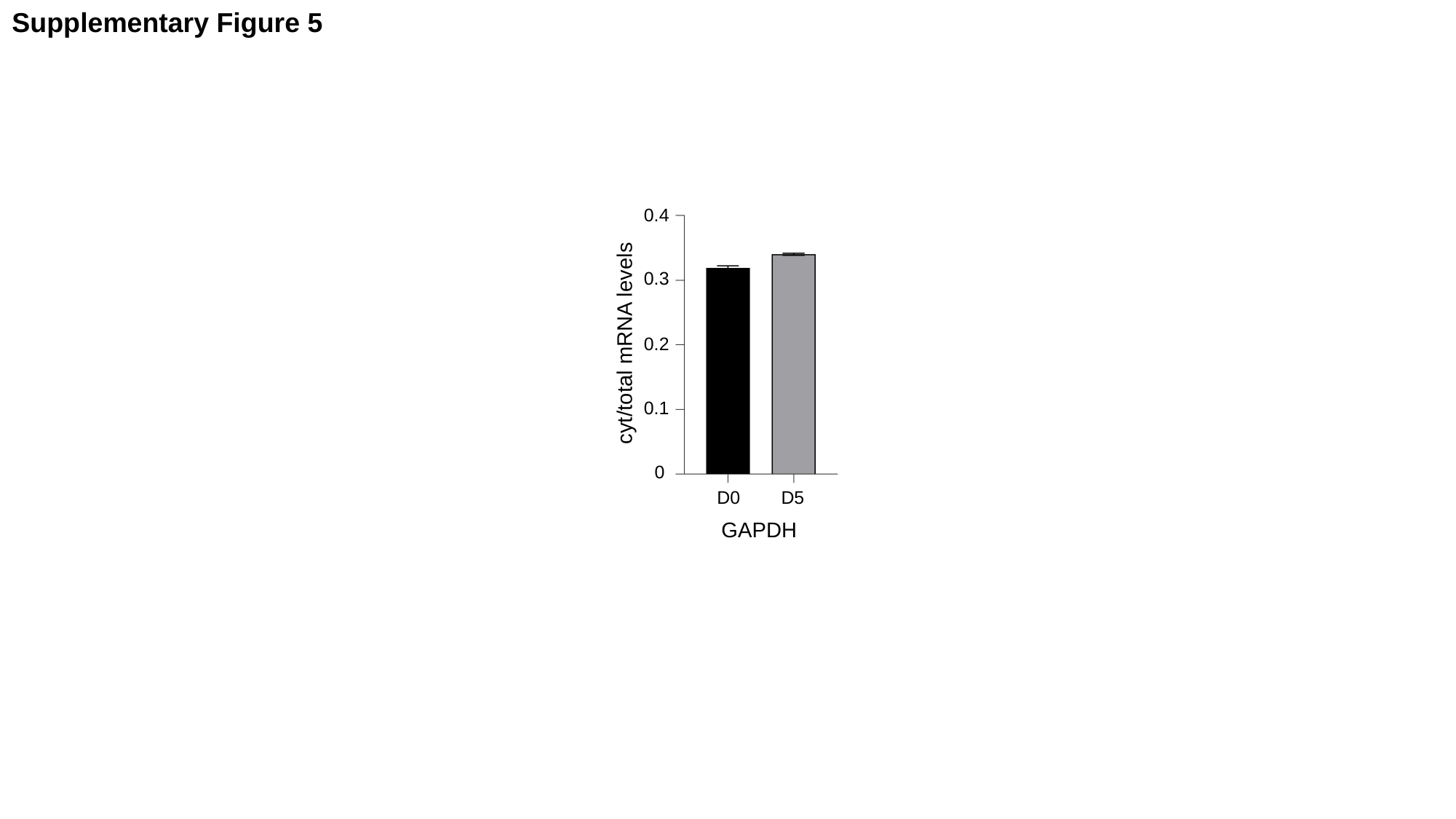

Supplementary Figure 5
0.4
0.3
cyt/total mRNA levels
0.2
0.1
0
D0
D5
GAPDH

## Slide 6
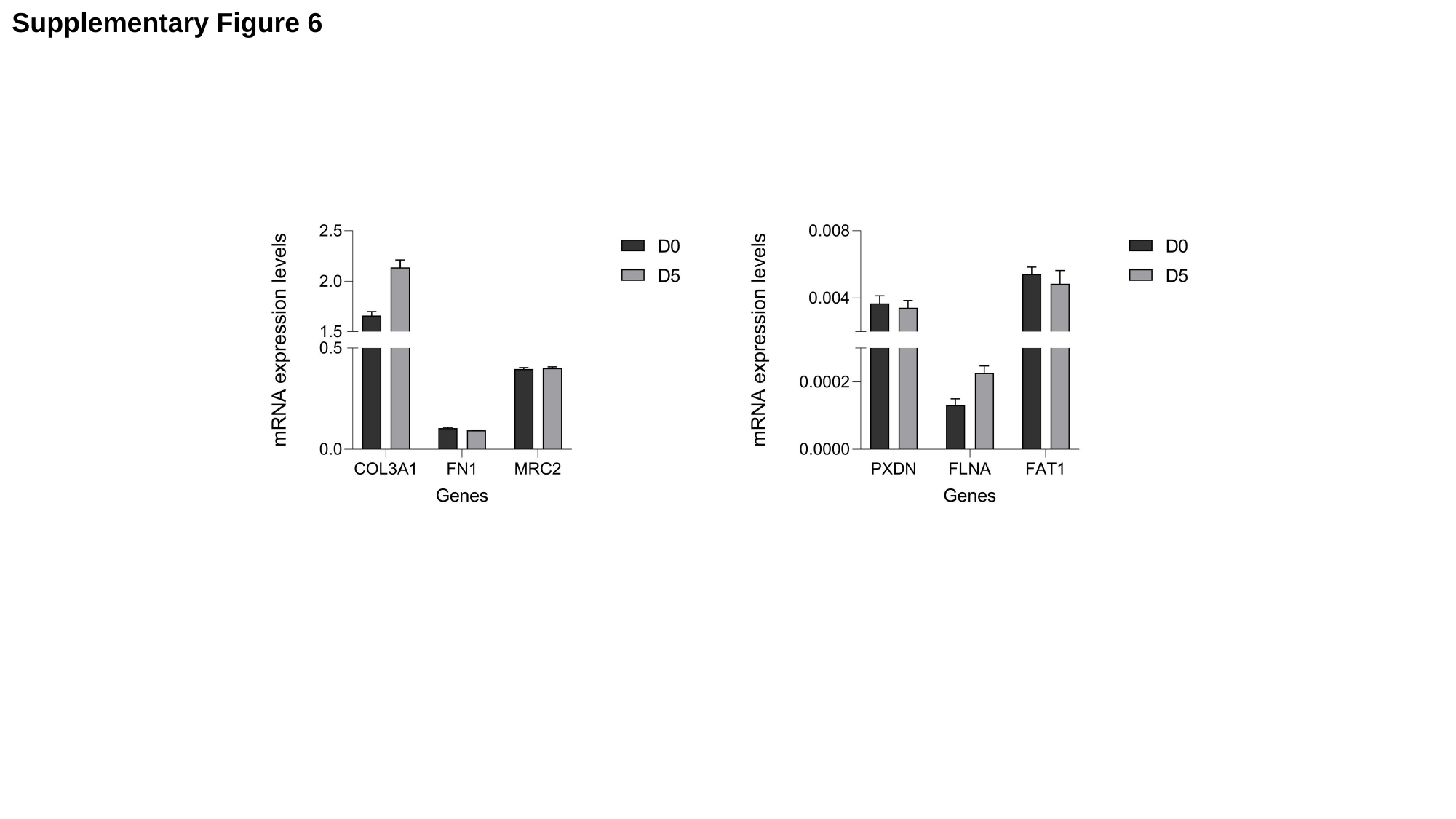

Supplementary Figure 6

## Slide 7
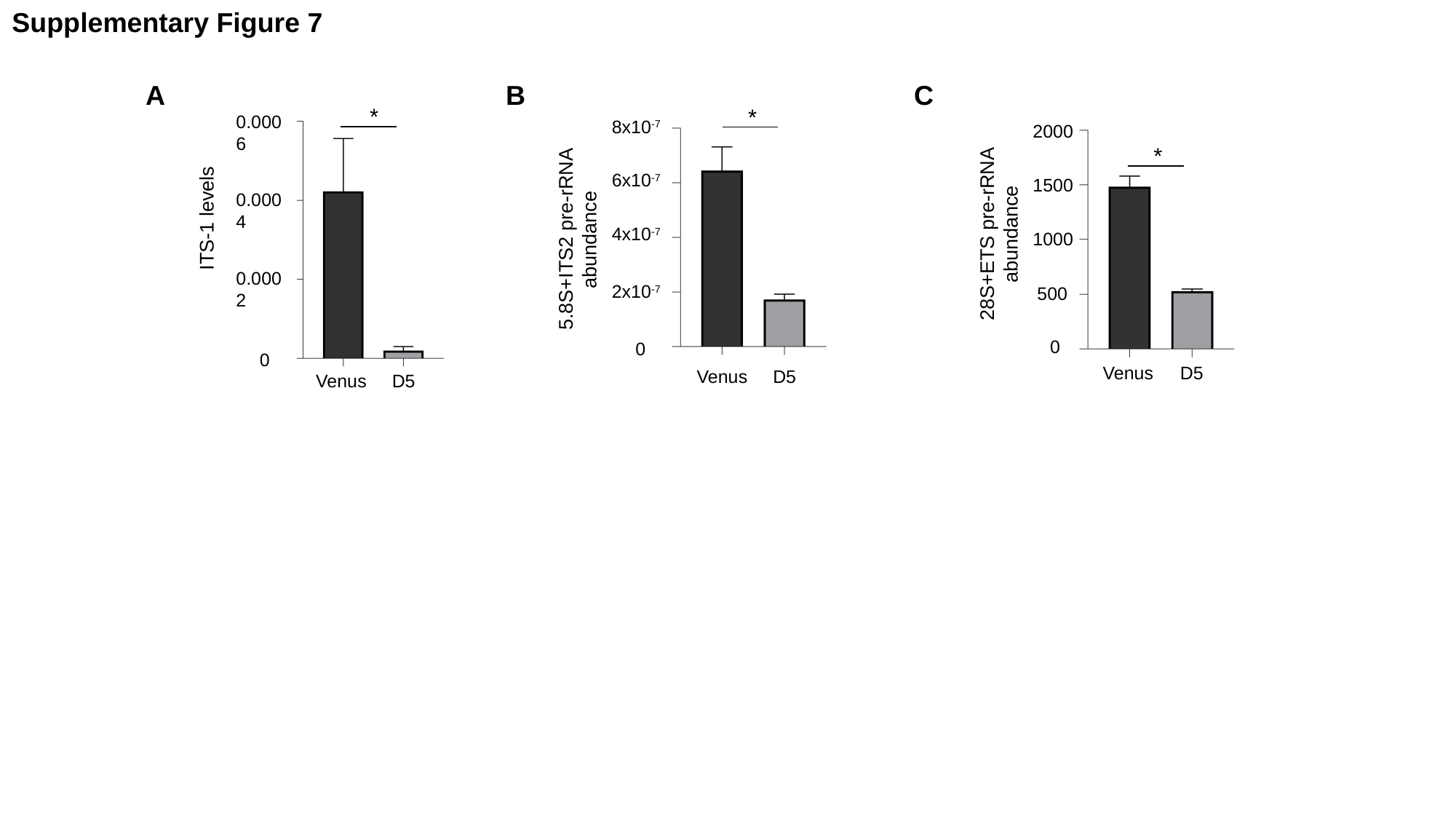

Supplementary Figure 7
A
B
C
0.0006
0.0004
ITS-1 levels
0.0002
0
Venus
D5
*
*
8x10^-7^
6x10^-7^
5.8S+ITS2 pre-rRNA abundance
4x10^-7^
2x10^-7^
0
Venus
D5
2000
*
1500
28S+ETS pre-rRNA abundance
1000
500
0
Venus
D5

## Slide 8
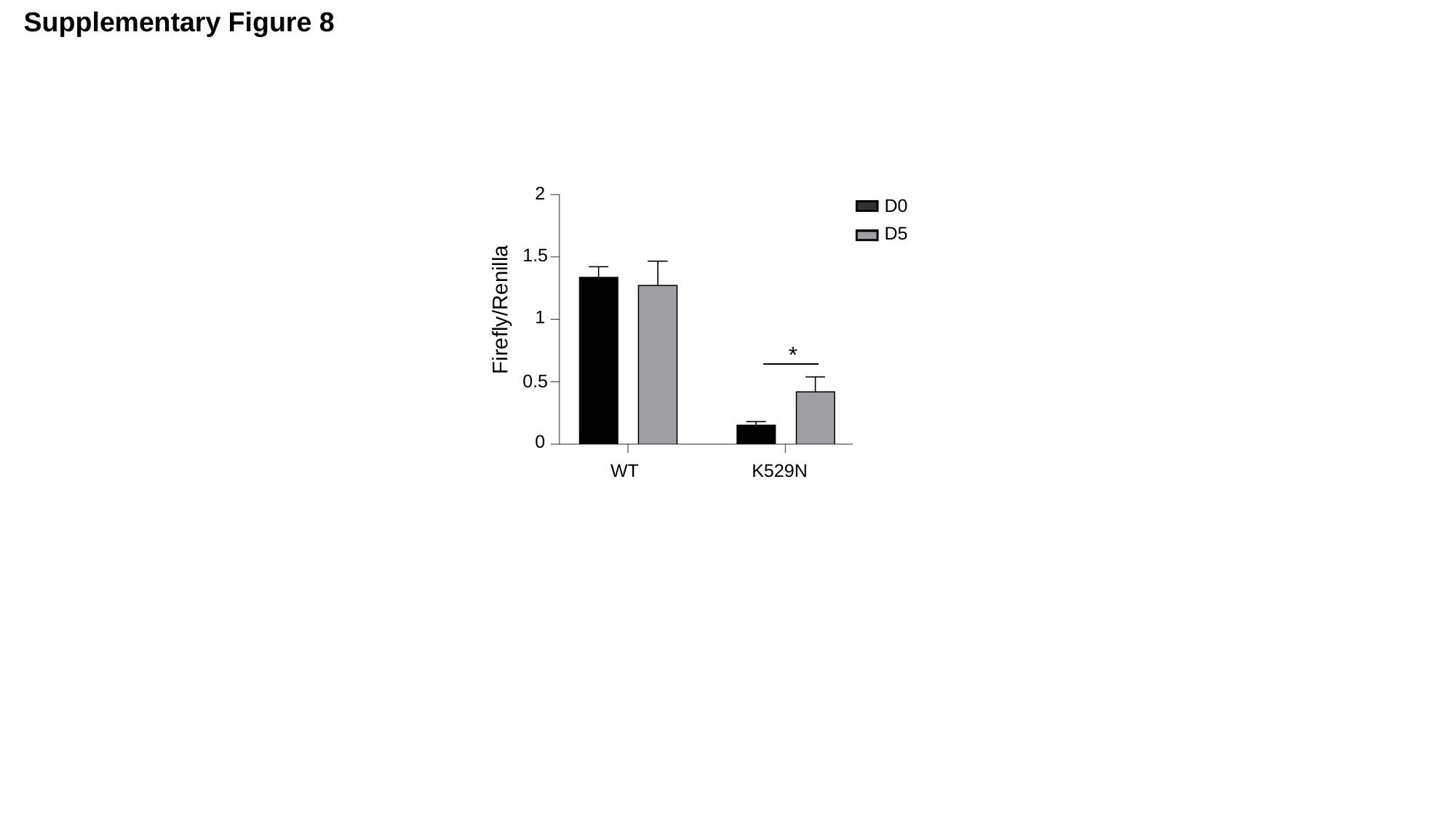

Supplementary Figure 8
2
D0
D5
1.5
Firefly/Renilla
1
*
0.5
0
WT
K529N

## Slide 9
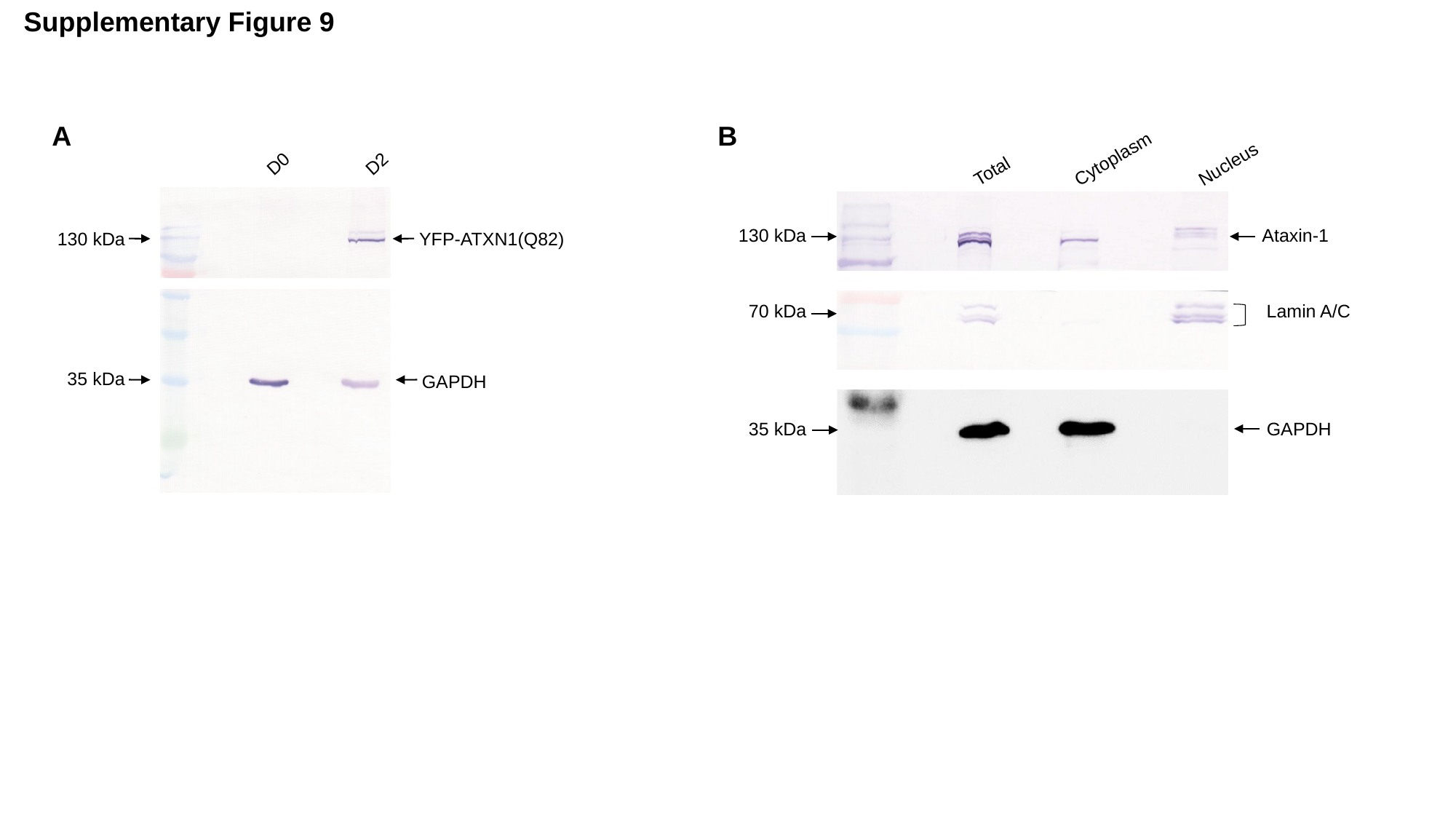

Supplementary Figure 9
A
B
Cytoplasm
Nucleus
Total
130 kDa
Ataxin-1
70 kDa
Lamin A/C
35 kDa
GAPDH
D0
D2
130 kDa
YFP-ATXN1(Q82)
35 kDa
GAPDH
